# Eliciting guilt in virtual reality games: interplay of self-attribution, presence, and morality

**DOI:** 10.3389/fpsyg.2024.1416258

**Published:** 2024-07-15

**Authors:** Changhyun Ahn, Ghee Young Noh

**Affiliations:** Media School, Hallym University, Chuncheon, Republic of Korea

**Keywords:** virtual reality, presence, morality, self-attribution, guilt, disposition theory

## Abstract

**Introduction:**

This study investigates the psychological mechanisms in virtual reality (VR) games, focusing on the interplay between character morality, self-attribution, presence, guilt, and their collective impact on player enjoyment. Based on Affective Disposition Theory, it hypothesizes that players’ moral judgments of characters significantly affect their engagement and enjoyment of VR narratives.

**Methods:**

A post-test between-subjects experiment was conducted with 97 participants to examine the influence of character morality on guilt through the mediation of self-attribution, and how these factors affect players’ sense of presence and overall enjoyment in VR games.

**Results:**

The findings indicate that self-attribution significantly mediates the relationship between character morality and guilt. Additionally, the sense of presence enhances enjoyment, with a stronger sense of ‘being there’ amplifying the emotional impact of players’ moral decisions.

**Discussion:**

This study highlights the full mediating effect of self-attribution in the context of VR gaming, intensifying players’ emotional responses to moral dilemmas. The results suggest that VR game designers should consider the moral implications of game narratives and character actions to create more emotionally engaging and ethically reflective gaming experiences. These insights have significant implications for VR game design and ethics, promoting greater ethical sensitivity among players.

## 1 Introduction

The evolution of virtual reality (VR) devices has significantly broadened the horizon of media consumption, particularly within the domain of video gaming. This immersive medium offers unparalleled depth to the gaming experience, potentially altering players’ psychological processes in unique and profound ways. Our investigation pivots on the intricate relationships among character morality, self-attribution, presence, guilt, and their collective influence on enjoyment in virtual reality gaming environments. Grounded in the theoretical bedrock of the Affective Disposition Theory (ADT) posited by Zillmann and Cantor (1976), this study delves into how players’ moral judgments about characters influence their enjoyment and engagement with media narratives.

The immersive nature of virtual reality games necessitates a nuanced understanding of how players interact with and perceive their virtual environments. Unlike traditional media, virtual reality’s participatory and immersive characteristics compel players to actively engage in the game’s narrative, making decisions that directly affect the outcomes. This level of engagement raises pertinent questions regarding the psychological dynamics of player experiences, particularly in relation to self-attribution, a process through which individuals ascribe actions and outcomes to themselves. With alignment between player actions and avatar movements considerably enhanced in virtual reality, the effects of self-attribution extend to morality, guilt, and presence, significantly affecting the overall game experience.

Building upon existing research by Ahn et al. (2021), our study explores these dynamics within virtual reality gaming, hypothesizing that self-attribution acts as a mediating factor between players’ moral judgments and their perceived sense of presence. This, in turn, influences their experience of guilt and enjoyment. Through the innovative integration of ADT with the concept of presence in virtual reality, we aim to shed light on the complex interplay between these factors, offering a novel perspective on the psychological processes underpinning player experiences in immersive gaming environments.

The current study examines the effects of character morality on guilt mediated by self-attribution. This current study also explores how these factors collectively influence players’ sense of presence and enjoyment, in order to contribute to a better understanding of virtual reality gaming psychology. The unique immersive capabilities of virtual reality not only heighten players’ sense of presence, but also amplify the emotional and cognitive impacts of moral decisions, potentially deepening the experience of guilt or moral satisfaction. As virtual reality continues to redefine the digital entertainment landscape, understanding these psychological dynamics has become crucial for both theoretical exploration and practical application in game design.

## 2 Influence of character morality on guilt

In exploring the interplay of moral perceptions and emotional reactions within virtual reality gaming, this study spotlights guilt as a core emotional response. This exploration is rooted in Zillmann and Cantor (1976) Affective Disposition Theory (ADT), which frames media enjoyment along a spectrum of emotional dispositions. ADT suggests that audiences continuously assess the morality of characters, which influences their enjoyment levels.

Building upon existing research, our study aims to understand the psychological dimensions of player experiences in virtual reality gaming, particularly focusing on the concept of guilt. It has been consistently observed that the implications of real world moral judgments are similarly observed in virtual settings (O'Donnell, 2005; Klimmt et al., 2006).

Multiple variables can be seen to influence the emotional weight of guilt. When players navigate through a game using avatars that engage in immoral activities, the sensation of guilt is heightened, compared to engagement in morally sound actions (Hartmann et al., 2010; Hartmann and Vorderer, 2010). Additionally, guilt is amplified when players commit acts of aggression toward Non-Playable Characters (NPCs) with human characteristics, compared to dehumanized NPCs (Lin, 2011). Moreover, guilt is more likely to be experienced when engaging in actions that are ethically questionable (Mahood and Hanus, 2017).

Although the virtual reality context adds a new layer to the research, we hypothesize that the essential psychological mechanisms will remain consistent, whether in a virtual reality setting or in a traditional game. Thus, we propose the following predictive hypotheses:

*Hypothesis* 1: Virtual reality character portrayals (manipulated moral vs. immoral) positively predict the perceived morality of virtual reality characters.

*Hypothesis* 2: The perceived morality of the virtual reality avatar controlled by the player will significantly and negatively predict the level of guilt experienced.

## 3 Self-attribution and morality

Self-attribution’s linkage to guilt and morality within gaming environments has been a focal point of research, revealing that gamers’ actions, when morally ambiguous or outright immoral, can spur feelings of guilt, with self-attribution acting as a pivotal mediator in this dynamic (Hartmann and Vorderer, 2010; Ahn et al., 2021). The way players perceive their in-game avatar’s morality significantly influences their self-attribution process. Engaging with avatars depicted as lacking moral integrity tends to elevate self-attribution for the avatar’s behaviors, conversely affecting the experience of guilt (Ahn et al., 2021).

The notion of identity within virtual realms has also been explored, indicating that players can adjust their self-concepts to align more closely with their avatars, blurring the lines between self and character (Conway et al., 2014; Nachez and Schmoll, 2022; İskender, 2023). This melding of gamer and avatar identities allows for a complex relationship between an individual’s real-world persona and their virtual counterpart, enabling a nuanced interplay of attributions toward both (Banks, 2015; Grizzard and Ahn, 2017).

Drawing from the principles of self-serving bias and attribution theory, individuals typically attribute successes to internal factors and failures to external ones (Heider, 1958; Kelley, 1967; Miller and Ross, 1975). In gaming contexts, this bias facilitates selective attribution, where morally upright actions are credited to the player, and morally dubious actions are attributed to the game or avatar (Bartel, 2015).

Bartel (2015) critically examined this dynamic by delving into the complex interplay between players and their avatars, particularly in games featuring morally complex characters, such as Trevor from Grand Theft Auto V. Despite engaging in morally reprehensible acts within the game, players find enjoyment in such scenarios, suggesting a more intricate connection between the player’s self-concept and the avatar’s actions. This scenario posits that the attribution of moral or immoral actions is not static, but fluctuates based on the character’s morality and the current situation. Such a fluid attribution mechanism underscores a sophisticated interaction between players’ moral assessments and their gaming experience, challenging simpler dichotomies of character-player identity, and pointing toward a more complex, situational attribution model (Weiner, 2018).

Building upon Bartel’s discussion and attribution theory, this study probes deeper into the self-attribution effects in virtual reality gaming. By utilizing virtual reality, which inherently enhances player-avatar congruence, we anticipate a more pronounced interplay between moral decision-making and guilt. This research aims to not only explore whether self-attribution serves as a mediator between character morality and guilt, but also to pave the way for investigating the intricate link between self-attribution and the sense of presence in virtual reality environments. Thus, the following hypotheses were formulated:

*Hypothesis* 3: The perceived morality of a virtual reality avatar controlled by a player will positively predict self-attribution.

*Hypothesis* 4: Self-attribution will positively predict guilt.

*Hypothesis* 5: Perceived morality of virtual reality avatars mediates the relationship between character portrayal and guilt.

*Hypothesis* 6: Perceived morality of virtual reality avatars and self-attribution serially mediate the relationship between character portrayal and guilt.

## 4 Self-attribution and presence in virtual reality

The interconnection between self-attribution and the feeling of presence has been the subject of extensive investigation in the field of virtual reality (VR). A cornerstone of this research is the principle demonstrated by the ‘rubber hand illusion,’ an experiment that reveals the brain’s proclivity to merge tactile and visual inputs to create the illusion of ownership over a fake limb, as described by Botvinick and Cohen (1998). This foundational study, while not VR-specific, elucidates the cognitive dissonance between proprioceptive feedback and visual cues. It serves as an analog for virtual reality scenarios, where users may attribute experiences mediated through avatars to themselves, thereby enhancing their sense of presence in the virtual environment (Morganti et al., 2009).

This illusion, which prioritizes visual perception over proprioception, tricks the mind into feeling a brushstroke on a rubber limb as if it were the participant’s actual limb. This psychological mechanism, emphasizing the power of sight over physical sensation, suggests that what we see can redefine our sense of body ownership (Botvinick and Cohen, 1998; Haans and IJsselsteijn, 2007). When this visual dominance occurs, the occipital lobe’s visual processing can impact the premotor cortex, inducing a self-attribution effect in which individuals attribute the artificial limb to themselves (Zeller et al., 2016).

Kanayama et al. (2021) extended this phenomenon to the virtual reality domain, confirming through EEG studies that virtual avatars could evoke similar illusions, indicating that visual stimuli alone can influence users to attribute parts of an avatar’s body to themselves. The sense of ownership and agency within virtual reality experiences, as elucidated by Kilteni et al. (2012), expands upon the rubber hand illusion. These sensations are crucial for a convincing sense of self-attribution and embodiment in virtual reality, which is further supported by empirical studies such as that of Jicol et al. (2021), which show that an increased sense of ownership can significantly enhance presence in virtual environments.

In light of the exploration of self-attribution and its relationship with morality and guilt, the dynamics of presence in virtual reality presented here gain an added layer of complexity. The mechanisms underpinning presence—notably illustrated by the ‘rubber hand illusion’ and its extension into virtual environments—underscore how virtual reality technology not only enhances the feeling of being physically and emotionally immersed, but also intensifies the psychological impacts of moral decisions made within these environments. This integration of self-attribution with the sense of presence suggests that virtual reality can uniquely amplify the moral and ethical dimensions of gameplay, potentially deepening experiences of guilt and moral satisfaction. As players navigate through virtual reality games, their increased sense of presence, fueled by the cognitive effects of self-attribution, may lead to more profound reflections on their actions and consequences, both virtual and potentially extrapolated to real-life moral reasoning. Therefore, the intersection of self-attribution and presence in virtual reality not only offers a richer, more immersive gaming experience, but also provides a unique lens through which to examine the complex interplay of morality, identity, and emotional response within virtual worlds.

*Hypothesis* 7: Self-attribution will positively predict presence.

## 5 Theoretical foundations of enjoyment in virtual reality

### 5.1 Affective disposition theory

Affective Disposition Theory (ADT), introduced by Zillmann and Cantor (1976), explains the relationship between moral judgments cast on media characters and the overall enjoyment derived from media narratives. According to ADT, audiences continuously assess the morality of characters, forming positive or negative dispositions toward them (Zillmann and Bryant, 1975; Zillman and Cantor, 1977). Enjoyment comes from seeing positive events happen to characters we favor and negative events happen to characters we do not favor (Raney, 2011).

### 5.2 Extensions to ADT: Moral sanction theory

In 2000, Zillmann extended ADT into the Moral Sanction Theory of Delight and Repugnance (MST), elaborating on the process of moral judgments and how they contribute to disposition formation and revision. MST introduces the concept of anticipatory emotions, where the audience anticipates the outcomes of characters’ actions based on their moral judgments. This anticipation is followed by the evaluation of the action’s outcome concerning justice, leading to empathy or counterempathy. Thus, MST presents a more complex pathway from moral judgment to enjoyment than initially proposed by ADT (Zillmann, 2000 2013).

Anticipatory emotions involve the feelings of hope or dread that arise as the audience foresees potential outcomes for the characters. Justice evaluations then assess whether the outcomes align with moral expectations, influencing feelings of satisfaction or disappointment. Finally, empathy or counterempathy responses are generated, reflecting a deeper emotional engagement with the narrative (Raney, 2006 2011). These mediating events illustrate the nuanced psychological processes that connect moral judgments to media enjoyment. For instance, anticipatory emotions can heighten the viewer’s engagement with the narrative, as they emotionally prepare for the characters’ fates. Justice evaluations reinforce moral standards by assessing the fairness of the outcomes, thereby influencing the viewer’s emotional response. Empathy and counterempathy responses further deepen the connection to the narrative by aligning the viewer’s emotions with the perceived justice or injustice experienced by the characters (Raney and Janicke, 2013).

### 5.3 Application of ADT and MST to virtual reality gaming

Virtual reality gaming offers a unique context to examine ADT and MST due to its immersive nature. Unlike traditional media, virtual reality requires active participation and decision-making that directly impact the narrative and outcomes. This participatory aspect introduces several mediating events that complicate the pathway from moral judgment to enjoyment. Players’ self-attribution of actions within the game and the resultant sense of presence significantly influence the emotional weight of their moral decisions.

In VR, the alignment between players’ actions and avatars’ movements enhances the feeling of presence, or the illusion of ‘being there’ (Gaggioli et al., 2016). This alignment amplifies the emotional resonance of moral decisions, as players perceive their actions in the game as a reflection of their moral choices. Consequently, self-attribution becomes a pivotal mediator, shaping the gaming experience and intertwining moral evaluation with the visceral feeling of presence.

ADT and MST involve a series of mediating events, including anticipatory emotions, justice evaluations, and resultant empathy or counterempathy (Raney, 2011). In the immersive context of VR, these mediating events are intensified by the technology’s ability to create a more direct and embodied experience of moral decisions and their outcomes.

Our empirical findings support this theoretical framework. Specifically, the study demonstrates that players’ self-attribution of in-game actions significantly mediates the relationship between character morality and emotional responses such as guilt. Furthermore, the enhanced sense of presence in virtual reality amplifies these effects, leading to a more profound impact on overall enjoyment. This underscores the importance of both technological and psychological factors in shaping the unique gaming experiences provided by virtual reality (Makransky et al., 2017). The immersive environment of virtual reality, with its enhanced presence and realistic engagement, provides a unique opportunity to observe and understand the complex interplay of these mediating factors.

### 5.4 Effect on enjoyment: guilt vs. presence

Central to understanding the immersive experience of virtual reality gaming is the role of self-attribution in mediating between players’ moral judgments and presence within the context of virtual reality gaming. Self-attribution is particularly salient in virtual reality because of the technology’s ability to closely align players’ actions with those of their avatars (Gaggioli et al., 2016). This alignment not only enhances the feeling of presence, or the illusion of ‘being there,’ but also amplifies the emotional resonance of moral decisions made within the game. Consequently, when players perceive their actions in the game as a reflection of their moral choices, this not only impacts their sense of guilt or moral satisfaction but also influences their overall enjoyment of the experience. The immersive nature of virtual reality, therefore, turns self-attribution into a pivotal factor that shapes the gaming experience, intertwining moral evaluation with the visceral feeling of presence, and ultimately affecting how players derive enjoyment from their virtual engagements. This interplay suggests a complex feedback loop in which a sense of presence bolstered by self-attribution can intensify emotional responses to moral dilemmas, thereby influencing virtual reality gamers’ engagement and enjoyment of virtual reality.

Our empirical findings support this theoretical framework, demonstrating that players’ self-attribution of in-game actions significantly mediates the relationship between character morality and emotional responses such as guilt. Furthermore, the enhanced sense of presence in VR amplifies these effects, leading to a more profound impact on overall enjoyment (Makransky et al., 2017). This highlights the importance of considering both the technological and psychological factors that contribute to the unique gaming experiences provided by virtual reality. By examining the specific elements of virtual reality that enhance presence, such as 360-degree immersion, our study underscores the complex interplay of factors that contribute to the heightened emotional and cognitive engagement in virtual reality gaming.

Within the expansive sphere of virtual reality, the psychological concept of presence assumes a paramount role, encapsulating the essence of “being there” or the sensation of co-presence (Biocca et al., 2003). Often associated with the realism of a virtual environment, presence also encompasses the emotional connectivity experienced with virtual entities. Lombard and Ditton (1997) propose that presence goes beyond the simplistic notion of “being there,” arguing for a broader, multi-faceted conceptualization that includes sensory immersion, technological transparency, and the subjective experience of spatiality.

Re-defining presence as a “perceptual illusion of non-mediation” (Lombard and Ditton, 1997), researchers underscore the complexity of creating a truly immersive experience that transcends the awareness of mediating technology, akin to the unconscious assimilation of eyeglasses into one’s visual field. Extending this framework, Lee (2004) suggests that presence has always been a fundamental human experience, predating technological advancements. Presence is thereby reconstituted as a psychological state in which virtual objects are experienced with tangible realism (Lee, 2004).

Makransky et al. (2017) operationalized Lee (2004) holistic concept of presence, devising a survey tool tailored to virtual reality gaming experiences. Their instrument measures the subdimensions of presence—physical, social, and self—as experienced within a virtual reality context (Makransky et al., 2017). This tool was employed in the current study to examine the effect of presence on enjoyment, elucidating the symbiotic relationship between the two in virtual reality gaming environments.

Virtual reality distinguishes itself from traditional gaming and media consumption through its unparalleled immersive capabilities, fundamentally altering players’ experience and engagement. Features such as 360-degree immersion, interactive environments, and haptic feedback are pivotal in creating a compelling sense of presence that traditional platforms cannot replicate. These VR-specific features foster a deeper emotional and moral engagement by allowing players to experience a simulated reality that closely mirrors their physical actions and decisions. For instance, the ability to gaze 360 degrees in a virtual environment or interact with it in a manner that feels physically tangible enhances the authenticity of the virtual experience. This heightened realism makes moral choices and actions within the game feel more consequential, amplifying the impact of self-attribution on players’ emotional responses. The immersive nature of virtual reality not only entertains, but also engages players in a reflective dialog with their moral selves, pushing the boundaries of traditional media’s capacity to elicit such introspection. Consequently, virtual reality’s unique features enhance the sense of being ‘there’ and deepening the player’s connection to the virtual world, making every decision, action, and its moral implications more vivid and impactful on their overall enjoyment of the game.

In synthesizing the interplay among self-attribution, presence, and enjoyment within virtual reality, this study embarks on a novel theoretical exploration, charting a course through the less-traversed dimensions of media psychology. Traditional applications of ADT and presence have rarely been integrated into the nuanced process of self-attribution within the immersive confines of virtual reality. By examining how virtual reality’s distinct features such as 360-degree immersion and interactive environments not only enhance the feeling of presence, but also facilitate a more embodied process of moral judgment and self-attribution, this study attempts to bridge a significant gap in existing literature.

Virtual reality’s capacity to simulate real-world interactions in a controlled, yet deeply immersive manner provides a unique experimental setting for investigating complex psychological dynamics. For instance, interactive environments in virtual reality enable players to physically make decisions that carry moral weight, offering an unparalleled opportunity to study how such actions influence self-attribution and, by extension, the emotional and cognitive presence experienced by users. These immersive features of virtual reality serve not only as technological enhancements but also as pivotal tools for engendering a deeper, more personal engagement with virtual narratives and ethical dilemmas.

This pioneering approach seeks to unravel the intricate ways in which immersive virtual reality technology can amplify the psychological processes underpinning moral judgment, self-attribution, and presence, thereby setting a foundation for a more profound understanding of how these factors collectively shape enjoyment in virtual environments. As such, this study not only contributes to the theoretical discourse by proposing a novel integration of ADT and presence with self-attribution in virtual reality, but also invites further empirical investigation into these dynamic interactions. Through this, we aim to illuminate the unique potential of virtual reality in enhancing the psychological realism and emotional depth of gaming experiences, ultimately enriching the discourse on media psychology and interactive entertainment.

*Hypothesis* 8: Guilt will negatively predict enjoyment.

*Hypothesis* 9: Presence will positively predict enjoyment.

## 6 Methods

### 6.1 Participants

As the study period coincided with the ongoing COVID-19 pandemic, strict health protocols were implemented. All research staff wore face masks at all times, and the laboratory was outfitted with various safety measures, such as hand sanitization stations with digital temperature checks, aerosolized disinfectant sprays, alcohol wipes, and a stock of additional masks available for participants.

The study was conducted at a large private university in South Korea, comprising of 97 participants (*N* = 97) aged between 20 and 29 years (38 women, 59 men, *M_age_* = 22.36, *SD* = 2.29) between October and December 2021. Each participant received a gift certificate for their time and involvement. Ethical guidelines established by the American Psychological Association were adhered to throughout the study, and all experimental procedures were approved by the institutional review board.

### 6.2 Design and procedure

This study was designed as post-test-between subject experiment with character biographies (moral: *n* = 49 vs. immoral: *n* = 48) as an Independent Variable (IV). Participant recruitment was effectively performed using a combination of digital and physical methods. Digital outreach included posts in online forums and social media groups associated with the campus, whereas physical recruitment was supported by flyers distributed throughout the university campus. Potential participants were directed to a customized website to register for the study. This registration process involved clicking on a link or scanning a QR code from advertisements, after which they were asked to provide a contact method - either a mobile phone number or email address - to arrange their participation details. This contact information was used only to coordinate the study, and was securely destroyed after the experiment.

Each experimental session was conducted individually and lasted approximately 30 min to ensure a consistent and controlled environment for all participants. Upon arrival at the research facility, participants began the study by accessing a survey on a dedicated computer linked to an online platform. The research team thanked the participants for their participation and explained the informed consent process in accordance with the requirements of the Institutional Review Board. This stage included answering questions about the consent form, emphasizing the voluntary nature of participation, and reassuring participants that there were no penalties for withdrawing from the study.

Following informed consent, participants were randomly assigned to one of two conditions that included pre-game stimuli presenting different moral biographies of the character they controlled in the virtual reality game. These conditions were designed to investigate the effects of character narratives, specifically moral versus immoral biographies, on participants’ experiences of self-attribution, presence, morality, and guilt in a virtual reality setting.

The two pre-game stimuli, described as ‘PLAYER GOOD’ or ‘PLAYER BAD,’ provided a background for the avatar controlled by the participant, outlining their motivations, ethical stance, and goals within the game’s universe. This setup aimed to provide participants with a moral framework that could influence their decisions and perceptions in the game.

Participants then engaged in the virtual reality game using an Oculus VR headset and controllers, immersing themselves in a scenario that corresponded to their pre-game stimulus. The immersive experience was designed to last for a standardized amount of time, allowing for consistent exposure to the game environment (5 min of gameplay) across participants. At the end of the gaming session, the participants were asked to complete a follow-up survey to assess the impact of the character biography on their reported levels of self-attribution, presence, perceived character morality, guilt, and enjoyment. Finally, the participants were debriefed about the aims of the study, thanked for their contributions, and compensated with a cultural voucher and new disposable mask.

### 6.3 Character biography (pre-game stimulus)

Before initiating the gameplay, participants were randomly allocated to engage with one of the two pre-game stimuli. These stimuli were designed to introduce the backstory of the avatar under the participant’s control, and set the stage for the game’s mission. The objective was to influence the participants’ perception of and approach to the game through these narratives, labeled PLAYER GOOD and PLAYER BAD.

In PLAYER GOOD condition, participants read the following narrative: “In this game you will play the role of a Special Forces Operative. You’re a SWAT officer. Your organization protects the vulnerable, fights crime and serves the public. The objective is to subdue drug dealers in Montoya, Colombia and secure evidence against them. You must use all available means to bring them to police justice.”

In PLAYER BAD condition, participants read the following narrative: “In this game you will play the role of a mercenary working for a private military company. You are a hired mercenary. Your organization despises the weak, ignores crime, and only looks out for corporate interests. The objective is to raid drug dealers in Montoya, Colombia, to loot and rob them of their supplies. You must secure profits by any means possible.”

**Figure 1:**
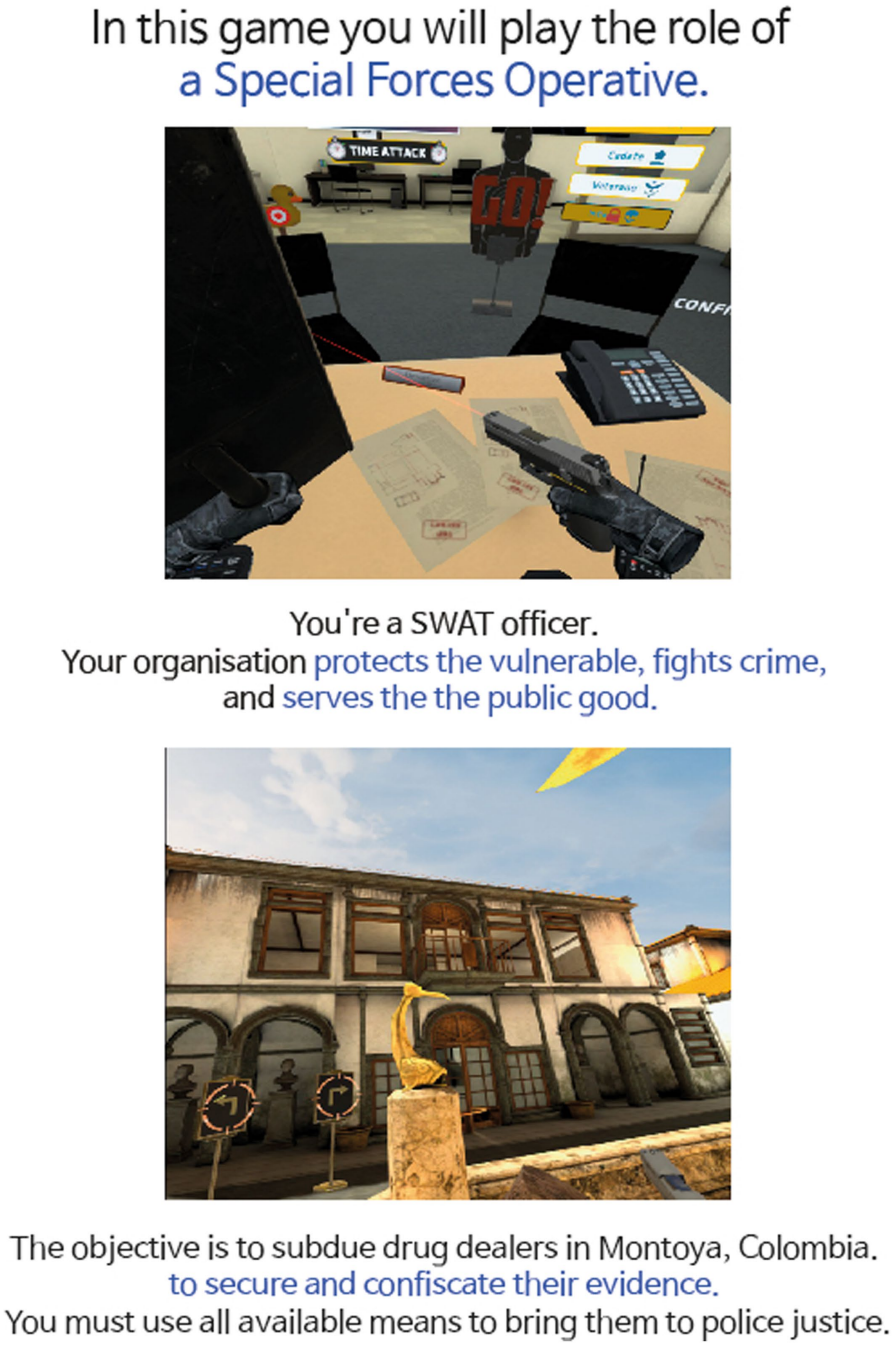
Pre-game stimulus of PLAYER GOOD condition. Adapted with permission from David Cano (Sumalab, Principal software engineer, developer of Crisis VRigade 2) http://www.sumalab.com.

**Figure 2:**
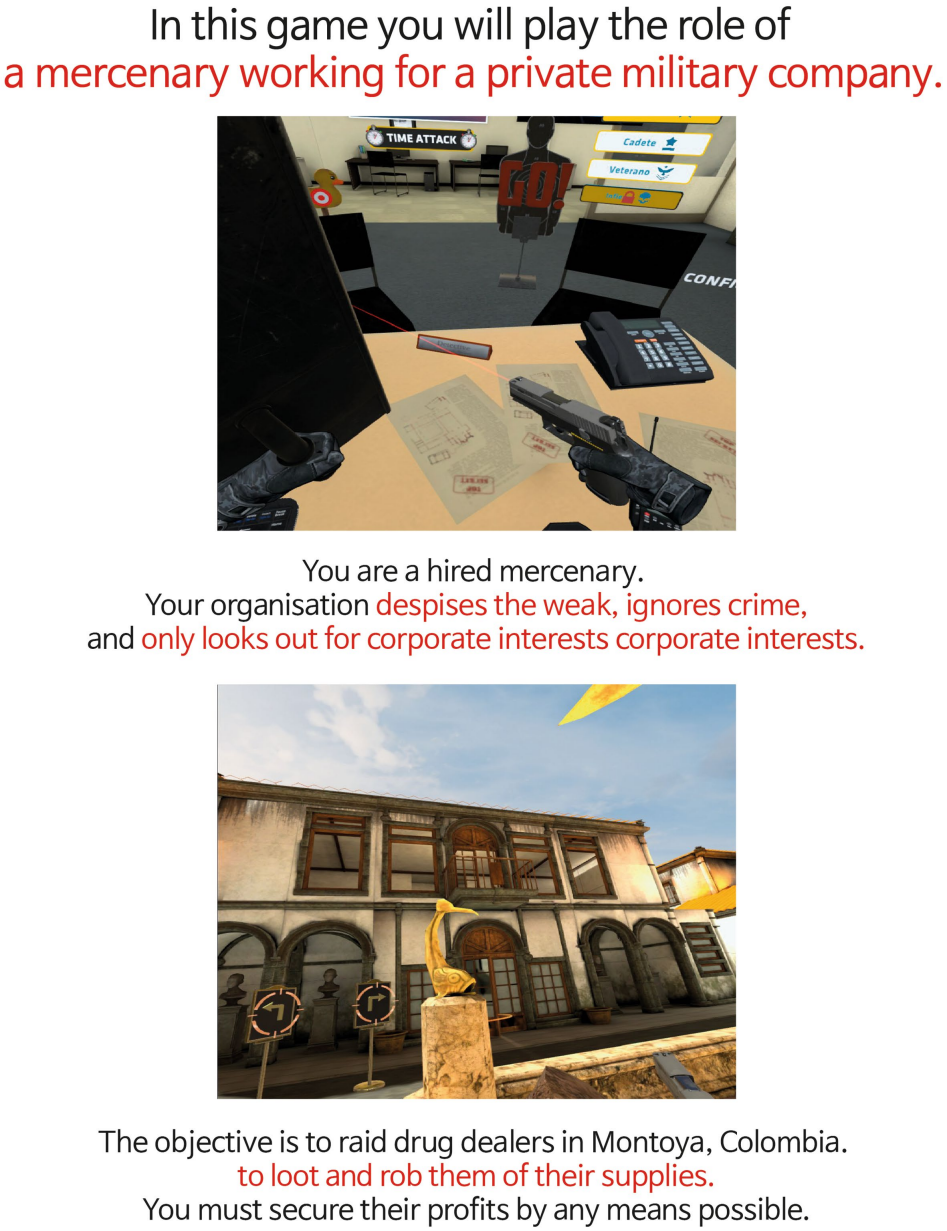
Pre-game stimulus of PLAYER BAD condition. Adapted with permission from David Cano (Sumalab, Principal software engineer, developer of Crisis VRigade 2) http://www.sumalab.com.

Each pre-game stimulus was crafted to be linguistically parallel in Korean, with the narratives structured to be approximately equal in length (PLAYER GOOD: 38 words; PLAYER BAD: 39 words), ensuring that both conditions offered comparable narrative context and complexity (Figures 1, 2).

### 6.4 Stimulus

In this study, “Crisis Brigade 2: Reloaded” for Oculus Quest 1 was selected as the stimulus—a first-person virtual reality shooter game praised for its intuitive gameplay, where players use the left arm controller to block bullets with a virtual shield, and the right arm controller to fire guns. The mission “Montoya” from the single-player campaign was specifically used. The choice of this game was based on several considerations: its straightforward blocking and firing mechanics reduce the skill gap between novice and seasoned virtual reality gamers; the in-game avatar’s minimalist design—showing only hands, a shield, and a gun—minimizes moral bias from physical appearance, consistent with research by Raney (2004) and Grizzard et al. (2018); and unlike the study by Hartmann and Vorderer (2010), which used UN soldier and terrorist avatars to delineate moral dichotomies, the enemies in “Crisis Brigade 2: Reloaded” are depicted in civilian clothes, blurring factional lines and eschewing clear moral distinctions.

**Figure 3:**
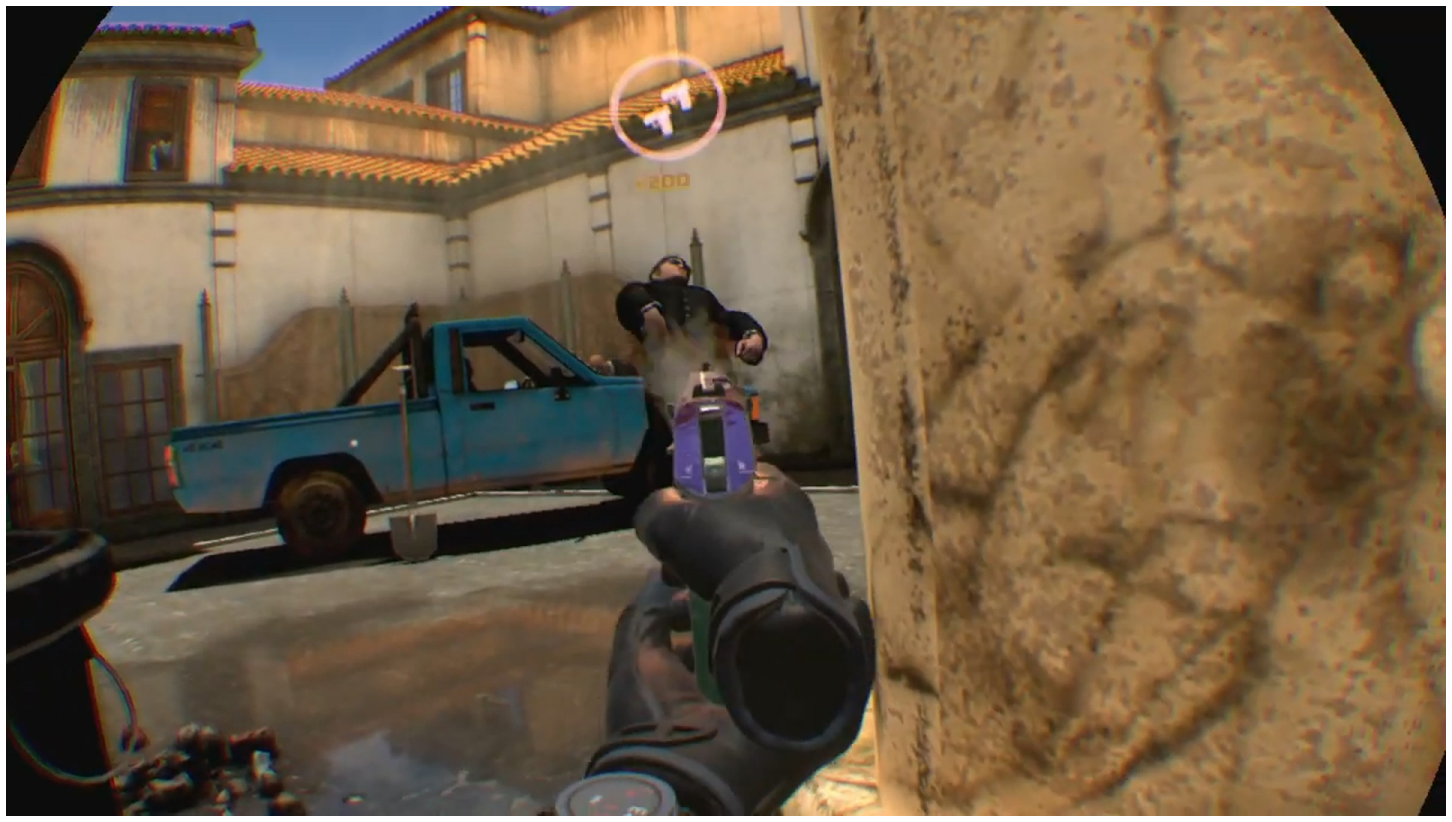
In-game screen shot of “Crisis Brigade 2: Reloaded” - “Montoya” mission. Adapted with permission from David Cano (Sumalab, Principal software engineer, developer of Crisis VRigade 2) http://www.sumalab.com.

To ensure internal validity, as recommended by Grizzard and Ahn (2017), the study manipulated character backstories rather than the game itself, meaning that all participants, regardless of being assigned to the PLAYER GOOD or PLAYER BAD condition, engaged with identical game content for 5 min. Additionally, to cater to the participants, all of whom were Korean, the game audio was switched from English to Spanish. This adjustment aimed to obscure the game’s original radio transmission, which could suggest that the avatar was a UN Special Force officer, thereby maintaining the neutrality of the avatar’s moral alignment (Figure 3).

#### 6.4.1 Permission to reuse and copyright

Figures, tables, and images will be published under a Creative Commons CC-BY license, and permission must be obtained for the use of copyrighted material from other sources (including republished/adapted/modified/partial figures and images from the Internet). It is the responsibility of the authors to acquire licenses, follow any citation instructions requested by third-party rights holders, and cover supplementary charges.

### 6.5 Apparatus

The virtual reality setup used in this study consisted of an Oculus Quest (1st generation) headset with its default controllers. The Oculus Quest is a standalone VR headset, meaning it operates independently without requiring a connection to an external computer. The device features Fresnel lenses and a display resolution of 1440×1600 per eye, with a refresh rate of 72 Hz. It provides six degrees of freedom (6 DoF) inside-out tracking via four integrated cameras. The headset weighs 571 grams and uses two Oculus Touch controllers (second generation), each weighing 135 grams and equipped with capacitive sensors for partial finger and thumb tracking. Detailed specifications of the Oculus Quest can be found at VR-Compare. This setup ensured participants had a seamless and immersive virtual reality experience.

### 6.6 Measures

*Character biography* was dummy-coded (1 = moral, 0 = immoral).

*Perceived morality of game character* was measured using 5-items and a 7-point Likert scale (0 = *not at all*, 7 = *very much so*), adapted from prior studies (Eden et al., 2015; Grizzard et al., 2018) like “good,” “heroic,” and reverse-coded “violent,” “aggressive,” “villainous.” Before creating a composite, a confirmatory factor analysis (CFA) was conducted. After dropping a single item (“violent”), CFA on the four items resulted in an acceptable fit: χ^2^(df = 2) = 19.531, *p* < 0.001, CFI = 0.93, RMSEA = 0.30, SRMR = 0.07. Based on model fit, a composite was created by averaging across the four items after reliability testing (α = 0.89, ω = 0.90, *M* = 3.86, *SD* = 1.42). An independent samples *t*-test revealed significant moral perception differences between conditions: the moral condition scored higher (*M* = 4.68, *SD* = 0.88) than the immoral condition (*M* = 3.02, *SD* = 1.39), *t*(79.17) = 7.03, *p* < 0.001, with a very large effect size (Cohen’s *d* = 1.43, see Sawilowsky, 2009), indicating the effective manipulation of character morality perception.

*Self-attribution* was measured using 4-items and a 7-point Likert scale (0 = *not at all*, 7 = *very much so*) adapted from previous research (Ahn et al., 2021), such as: “The actions my avatar committed in the VR game represent myself,” or “I felt responsible for the actions that I did in the VR game.” A CFA on the items resulted in an excellent fit: χ^2^(df = 2) = 1.35, *p* < 0.001, CFI = 1.00, RMSEA = 0.00, SRMR = 0.02. Based on model fit, a composite was created by averaging across the four items after reliability testing (*α* = 0.78, *ω* = 0.81, *M* = 4.44, *SD* = 1.45).

*Presence* was measured by 15-items and a 5-point Likert scale (0 = *not at all*, 5 = *very much so*) from Makransky et al. (2017). This scale included scales of physical presence (e.g., “the VR environment seemed real to me”), social presence (e.g., “I felt like I was in the presence of another person in the VR environment”), and self-presence (e.g., “I felt like my real hand was inside of the VR environment”). A CFA on the items resulted in an acceptable fit after dropping two items (*physical presence:* “I had a sense of acting in the virtual environment, rather than operating something from outside,” *self-presence:* “I felt like my real arm was projected into the virtual environment through my virtual embodiment), χ^2^(df = 62) = 90.42, *p* < 0.05, CFI = 0.96, RMSEA = 0.07, SRMR = 0.04. Based on model fit, a composite was created by averaging across the 13 items after reliability testing (*α* = 0.93, *ω* = 0.94, *M* = 3.39, *SD* = 0.90).

*Guilt* was measured using 6-items and a 7-point Likert scale (0 = *not at all*, 7 = *very much so*), adapting from previous studies (Hartmann et al., 2010; Hartmann and Vorderer, 2010; Ahn et al., 2021) such as (*feeling*) “guilty,” “regret,” “sorry for what I did,” “feeling like doing something wrong,” “shameful,” and “blameworthy.” A CFA on the items resulted in an excellent fit: χ^2^(df = 9) = 28.86, *p* < 0.01, CFI = 0.96, RMSEA = 0.15, SRMR = 0.04. Based on model fit, a composite was created by averaging across the six items after reliability testing (*α* = 0.93, *ω* = 0.93, *M* = 1.89, *SD* = 1.05).

*Enjoyment* was measured using 5-items on a 7-point Likert scale (0 = not at all, 7 = very much so), adapted from previous studies (Tauer and Harackiewicz, 1999; Hartmann and Vorderer, 2010): (*VR game was*) “very interesting,” “boring (reverse coded),” “fun,” “waste of time,” “enjoyable.” A CFA on the items resulted in an excellent fit: χ^2^(df = 5) = 6.97, *p* = 0.22, CFI = 0.99, RMSEA = 0.06, SRMR = 0.03. Based on the model fit, a composite was created by averaging the six items after reliability testing (*α* = 0.87, *ω* = 0.88, *M* = 4.76, *SD* = 0.40).

### 6.7 Analysis plan

**Figure 4:**
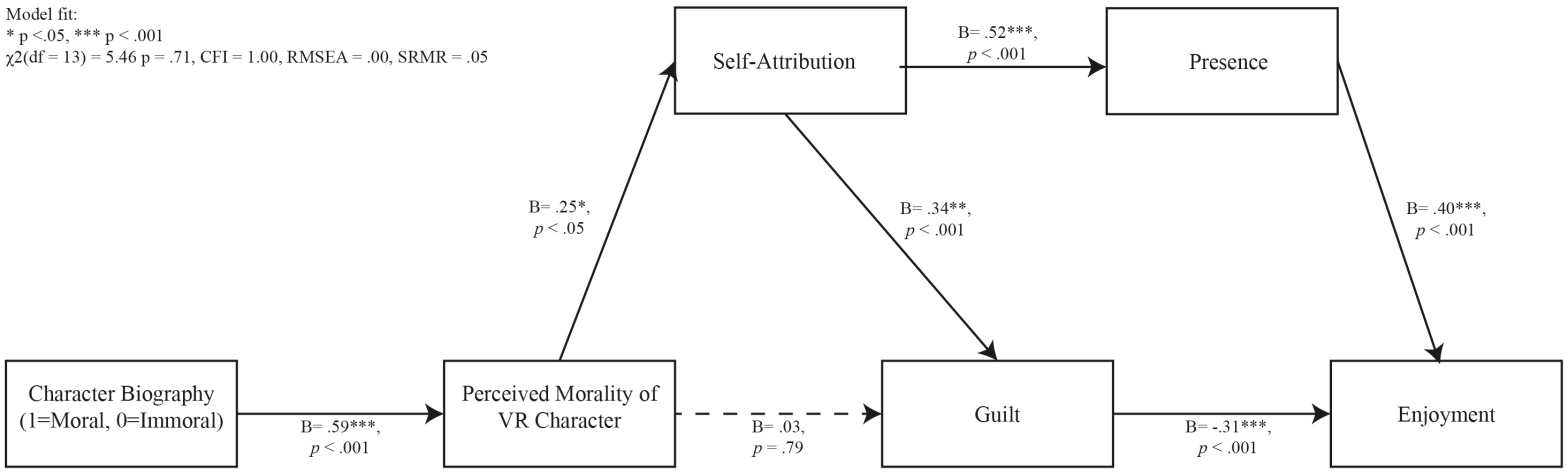
Path model results for hypotheses testings.

This study tested the hypotheses in a path model using AMOS with a maximum likelihood estimation. Specifically, we tested the following settings: bootstrap = 5,000 a 95% confidence interval (bias-corrected). Figure 4 shows the model fit and the results.

## 7 Results

### 7.1 Hypothesis testing

The study results were consistent with Hypothesis 1. Character biography (1: moral, 0: immoral) significantly and positively predicted the perceived morality of virtual reality characters (*B* = 0.59, *p* < 0.001). Participants who received the character biography of a SWAT officer significantly perceived that the character was more moral (*M* = 4.68, *SD* = 0.88) than participants who received the character biography of a hired mercenary (*M* = 3.02, *SD* = 1.39).

While the results were inconsistent with Hypothesis 2; the perceived morality of virtual reality characters did not significantly predict guilt (*B* = 0.03, *p* = 0.79), they were consistent with Hypothesis 3. The perceived morality of virtual reality characters significantly and positively predicted self-attribution (*B* = 0.25, *p* < 0.05); participants who perceived the virtual reality character as moral attributed their in-game actions to themselves.

Similarly, the results validated Hypothesis 4, revealing that self-attribution significantly and positively predicted guilt (*B* = 0.34, *p* < 0.001). Participants who attributed in-game actions to themselves more, demonstrated higher levels of guilt.

Meanwhile, although character biography significantly predicted the perceived morality of virtual reality characters (*B* = 0.59, *p* < 0.001), this perceived morality did not significantly predict guilt (*B* = 0.03, *p* = 0.79), hence disproving Hypothesis 5.

As for Hypothesis 6, character biography significantly predicted perceived morality of the virtual reality characters (*B* = 0.59, p < 0.001), which significantly positively predicted self-attribution (*B* = 0.25, *p* < 0.001), which predicted guilt (*B* = 0.34, *p* < 0.001).

The data showed results consistent with Hypothesis 7. Self-attribution significantly and positively predicted presence (*B* = 0.52, *p* < 0.001). Higher levels of presence were shown by participants who attributed themselves to their in-game actions.

The data showed results consistent with Hypothesis 8. Guilt significantly and negatively predicted presence (*B* = −0.31, *p* < 0.001). Participants who experienced a higher degree of guilt showed a lower degree of enjoyment.

These data are consistent with Hypothesis 9; presence significantly and positively predicted enjoyment (*B* = 0.40, *p* < 0.001), with participants who felt a higher degree of presence showing a higher degree of enjoyment.

### 7.2 Indirect effects

To further examine the serial indirect effects between variables, we employed a bootstrapping analysis with 5,000 bootstrap samples and 95% bias-corrected confidence intervals. The results, detailed in Table 1, explore the intricate dynamics between character biography, perceived morality of the virtual reality character, self-attribution, guilt, presence, and enjoyment.

**Table 1:** Standardized indirect effects (Bootstrap = 5,000, bias-corrected, 95% C.I.).

Path	Std. indirect effect	Boot. SE	Lower bound (BC)	Upper bound (BC)	*p* (Two-tailed)
Character biography to self-attribution	0.145	0.066	0.015	0.274	0.029
Character biography to guilt	0.065	0.054	−0.049	0.164	0.234
Character biography to presence	0.076	0.034	0.012	0.146	0.022
Perceived morality of game character to guilt	0.085	0.049	0.010	0.200	0.024
Perceived morality of VR character to presence	0.129	0.059	0.018	0.249	0.025
Self-attribution to enjoyment	0.104	0.067	−0.027	0.231	0.126

Consistent with Hypothesis 6, character biography indirectly influenced self-attribution significantly (*B* = 0.145, S.E. = 0.07, *p* < 0.05). Conversely, the total standardized indirect effects of character biography on guilt did not reach significance (*B* = 0.07, S.E. = 0.05, *p* = 0.23), suggesting a nuanced impact of character biography on guilt. Perceived mortality of the virtual reality character exerted a significant indirect effect on guilt (*B* = 0.09, S.E. = 0.05, *p* < 0.05). Given the non-significant direct effect of perceived morality on guilt (*B* = 0.03, *p* = 0.79), these results imply that self-attribution may serve as a full mediator between perceived morality and guilt in the context of virtual reality gaming.

The total standardized indirect effects of self-attribution on enjoyment were also not significant (*B* = 0.10, S.E. = 0.07, *p* = 0.13). However, a notable pathway was found from character biography through perceived morality and self-attribution to presence, which demonstrated a significant indirect effect (*B* = 0.08, S.E. = 0.03, *p* < 0.05). Furthermore, the pathway from perceived morality to self-attribution to presence was significant (*B* = 0.13, S.E. = 0.06, *p* < 0.05). While guilt negatively affected enjoyment directly (*B* = −0.31, *p* < 0.001), the overall indirect effect from character biography to guilt was not significant (*B* = 0.07, S.E. = 0.05, *p* = 0.23). Taken together, these results suggest that the pathway leading to presence from self-attribution wields a stronger influence than the psychological attribution of guilt stemming from self-attribution, particularly within the context of virtual reality gaming.

## 8 Discussion

### 8.1 Self-attribution’s full mediation and the dominance of presence in VR

Building on the conceptual framework established by Ahn et al. (2021) and Bartel (2015), this study advances our understanding of self-attribution processes and self-serving biases in the immersive context of virtual reality games. While Ahn et al. (2021) used a third-person perspective game to examine the effects of character morality on self-attribution and guilt, and found partial mediation, our study presents a first-person immersive experience through ‘Crisis Brigade 2,’ finding a full mediation effect. This shift is particularly noteworthy, highlighting the increased self-attribution to in-game actions induced by virtual reality technology in players, reinforcing the internalization of in-game actions, and leading players to attribute more positive outcomes to themselves and negative outcomes to external factors, even in the face of morally questionable in-game actions.

Our findings indicate a full mediation effect of self-attribution on the relationship between character morality and guilt in virtual reality. This contrasts with Ahn et al. (2021) study, which found only partial mediation. One potential explanation for this discrepancy is the enhanced immersive nature of VR. Kanayama et al. (2021) and Kilteni et al. (2012) suggest that VR’s immersive qualities can significantly strengthen the user’s sense of presence and self-attribution, leading to more profound emotional responses such as guilt.

In traditional digital games, players typically interact with the game environment through a 2D screen using controllers such as PlayStation or Xbox controllers, or keyboard and mouse setups. These types of games are characterized by a separation between the player’s physical movements and the avatar’s actions. The player remains seated and controls the avatar’s movements with their hands, which do not necessarily correspond to the avatar’s actual limb movements. Additionally, in traditional games, the player’s perspective may not always align with the avatar’s viewpoint, as seen in third-person games where players view their avatar from an external perspective. In Ahn et al. (2021), for example, the study involved players controlling avatars in a third-person perspective on a 2D screen using a PlayStation controller. This setup inherently creates a separation between the player and the avatar, leading to a weaker sense of self-attribution.

In contrast, virtual reality (VR) games provide a much more immersive experience by aligning the player’s physical movements with the avatar’s actions. In VR environments, the avatar’s movements mirror the player’s hand and body movements, and the visual perspective matches the player’s eye level and direction. This creates a seamless and convincing sense of agency, where players feel that their actions are directly translating into the virtual world, thereby intensifying emotional experiences. The alignment between the player’s physical actions and the avatar’s movements in VR can explain why self-attribution fully mediates the effect of character morality on guilt in our study, as opposed to the partial mediation observed in traditional digital gaming contexts.

Our findings suggest that despite the immersive qualities of virtual reality, which may increase self-serving bias, narrative elements such as character biographies continue to significantly influence perceived morality, self-attribution, and presence. The pathway from self-attribution to presence, and subsequently to enjoyment, carries more weight in virtual reality settings than the pathway from self-attribution to guilt. This highlights the heightened importance of presence in virtual reality environments, potentially altering well-established psychological processes, such as self-attribution bias.

The full mediation effect of self-attribution on the relationship between perceived character morality [unlike Ahn et al. (2021), which found partial mediation] and guilt in virtual reality suggests that players are likely to experience stronger emotional reactions when they fully attribute their actions to themselves. This finding is consistent with the immersive and personal nature of virtual reality games, and reflects a deeper psychological engagement with the virtual experiences afforded by this technology.

In light of these findings, future research should explore the boundary conditions of self-serving bias in different gaming modalities, including virtual reality. Given the central role of presence identified in our study, investigations into how virtual reality games enhance or diminish this sense should be prioritized. Such studies will further elucidate the complex interplay of narrative, technology, and psychological processes in video games, thereby informing game design and narrative structuring to optimize player engagement and satisfaction.

### 8.2 Self-attribution vs. character identification

To advance the study of player psychology in video games, particularly through the lens of virtual reality, it is important to distinguish between the concepts of self-attribution and character identification. While both consider how players relate to their in-game characters, they are conceptually distinct and play different roles in our understanding of player experience.

Self-attribution, the central construct in this study, refers to the degree to which players believe that their in-game actions reflect their personal values and choices. This is distinct from character identification, through which players empathize with or adopt a game character’s perspective. Although they may seem similar, self-attribution is particularly salient in virtual reality because of the immersive nature of the medium, which may lead players to more deeply internalize their in-game behavior.

Our current study extends the work of Ahn et al. (2021) by not only confirming the importance of self-attribution in traditional gaming contexts, where it was found to partially mediate the relationship between character actions and guilt, but also by demonstrating its enhanced role in virtual reality environments. Here, self-attribution fully mediates this relationship, suggesting a deeper connection between players’ perceived personal agency and their emotional responses in virtual reality gaming.

To further clarify, while research by Ahn et al. (2021) and the present work both utilized the self-attribution framework, the full mediation effect is observed in the novel virtual reality context. This distinction is important for reviewers who may question the apparent conceptual overlap with character identification. The finding of full mediation in virtual reality underscores the robustness of self-attribution as a concept for studying player experience, particularly how players reconcile their virtual actions with their self-concept in highly immersive gaming environments.

Our study clearly reveals self-attribution to be a crucial concept in unpacking the psychological nuances of gaming, offering a nuanced perspective on more traditional notions of character identification. This distinction, and the shift from partial to full mediation in virtual reality, provide a compelling narrative for the unique psychological effects of virtual reality gaming, and sets the stage for future research to explore these constructs in greater depth.

### 8.3 Practical implications for VR game design and ethics

The results of this study have significant practical implications for the design of virtual reality games. Full mediation through self-attribution highlights the need for game designers to create narratives and character biographies that foster the appropriate levels of moral engagement and responsibility. Designers should carefully consider the ethical dimensions of player actions in games, recognizing that the immersive nature of virtual reality can intensify players’ emotional responses to their in-game behavior.

The blurring of boundaries between the self and the avatar in virtual reality makes the narrative context and moral framing of characters even more powerful. Therefore, developers should implement mechanisms that allow players to reflect on the morality of their actions, perhaps through in-game consequences or reflective story elements, in order to cultivate a responsible gaming environment.

Furthermore, the strong effect of presence on enjoyment in virtual reality suggests the need to enhance the sense of ‘being there.’ Design features that promote presence, such as environmental interactivity, responsive storytelling, and intuitive control schemes can lead to more immersive and emotionally fulfilling gaming experiences. In doing so, game designers not only increase player satisfaction, but also potentially mitigate the negative emotional effects associated with guilt.

Ethically, the tendency toward a self-serving bias in virtual reality raises questions about the potential for desensitization to immoral actions. As players may attribute negative outcomes to external factors, content creators should be wary of normalizing negative behaviors and should consider implementing moral dilemmas that challenge them to engage more deeply with the consequences of their virtual actions.

In light of these considerations, virtual reality game developers have a unique opportunity to positively influence player psychology. Through thoughtful design, they can create experiences that encourage players to engage with content in an ethically aware, emotionally rich, and introspective manner, contributing to a broader discourse on the role of morality and ethics in digital entertainment.

## 9 Limitations

First, the study sample was largely homogeneous and drawn from a specific demographic group that may not have encapsulated the full spectrum of virtual reality gamers. Although our findings provide initial insights into the relationship between self-attribution and a heightened experience of presence in virtual reality, the extent to which they can be generalized across different populations remains an area for further exploration. Future research should aim to include a more diverse cohort, encompassing a broader age range, varying levels of gaming experience, and cultural backgrounds to confirm the universality of our conclusions.

Second, the singular focus on one virtual reality shooting game could limit the scope of our conclusions. Different virtual reality game genres and experiences may elicit different levels and aspects of presence and self-attribution, thereby influencing the emotional and psychological outcomes of gameplay. Future studies should thus aim to diversify the types of virtual reality content used, in order to broaden understanding of how immersive technology influences gamer experiences.

Finally, moral decision-making within the game was relatively simplistic, which may not accurately reflect the complexity and nuances of real-world ethical considerations. The moral choices presented were somewhat binary and did not allow for a full range of human moral reasoning. To address this, it would be beneficial for future research to design or select virtual reality experiences that offer more complex moral puzzles, requiring players to navigate a spectrum of moral choices. This approach could provide richer data and a deeper understanding of how morality interacts with presence and enjoyment in virtual environments.

## 10 Conclusion

Our research corroborates and extends the findings of Ahn et al. (2021), providing compelling evidence of the pervasive nature of self-attribution and the emergent importance of presence in shaping players’ emotional experiences in virtual reality. This contributes to a more nuanced understanding of player experiences in virtual reality, emphasizing the critical role of self-attribution across different gaming modalities, and the potential for immersive technologies to reshape established psychological processes.

In conclusion, the novel insights gained by comparing these two studies highlight the transformative potential of virtual reality technology on player psychology. The profound impact of the medium on players’ moral perceptions and emotions offers valuable implications for the ethical considerations of virtual reality content creators, game designs, and future research directions.

## Data availability statement

The raw data supporting the conclusions of this article will be made available by the authors, without undue reservation.

## Ethics statement

The studies involving humans were approved by Hallym University Institutional Review Board. The studies were conducted in accordance with the local legislation and institutional requirements. The participants provided their written informed consent to participate in this study.

## Author contributions

CA: Conceptualization, Formal analysis, Investigation, Methodology, Writing – original draft, Writing – review & editing. GN: Writing – original draft, Writing – review & editing.

## References

[ref1] Ahn C. Grizzard M. Lee S. (2021). How do video games elicit guilt in players? Linking character morality to guilt through a mediation analysis. Front. Psychol. 12, 1–6. doi: 10.3389/fpsyg.2021.666518, PMID: 34239481 PMC8258373

[ref2] Banks J. (2015). Object, me, Symbiote, other: a social typology of player-avatar relationships. First Monday 20:2. doi: 10.5210/fm.v20i2.5433

[ref3] Bartel C. (2015). Free will and moral responsibility in video games. Ethics Inf. Technol. 17, 285–293. doi: 10.1007/s10676-015-9383-8

[ref4] Biocca F. Harms C. Burgoon J. K. (2003). Toward a more robust theory and measure of social presence: review and suggested criteria. Presence: Teleoperators Virtual Environ. 12, 456–480. doi: 10.1162/105474603322761270

[ref5] Botvinick M. Cohen J. (1998). Rubber hands ‘feel’ touch that eyes see. Nature 391:756. doi: 10.1038/35784, PMID: 9486643

[ref6] Conway L. Gomez-Garibello C. Talwar V. (2014). Moving from traditional bullying to cyberbullying: the role of moral emotions and reasoning. Alberta J. Educ. Res. 60, 216–220. doi: 10.55016/ojs/ajer.v60i1.55899

[ref7] Eden A. Oliver M. B. Tamborini R. Limperos A. Woolley J. (2015). Perceptions of moral violations and personality traits among heroes and villains. Mass Commun. Soc. 18, 186–208. doi: 10.1080/15205436.2014.923462

[ref8] Gaggioli A. Ferscha A. Riva G. Dunne S. Viaud-Delmon I. (2016). Human computer confluence: Transforming human experience through symbiotic technologies, vol. 2016. Berlin, Germany: De Gruyter Open.

[ref9] Grizzard M. Ahn C. (2017). “Morality & personality: perfect and deviant selves” in Avatar, assembled: The social and technical anatomy of digital bodies. ed. Banks J. (New York: Peter Lang Publishing), 117–126.

[ref10] Grizzard M. Huang J. Fitzgerald K. Ahn C. Chu H. (2018). Sensing heroes and villains: character-schema and the disposition formation process. Commun. Res. 45, 479–501. doi: 10.1177/0093650217699934

[ref11] Haans A. IJsselsteijn W. A. (2007). “Self-attribution and telepresence”, in 10th Annual International Workshop on Presence (Barcelona, Spain: Starlab), 51–58.

[ref12] Hartmann T. Toz E. Brandon M. (2010). Just a game? Unjustified virtual violence produces guilt in empathetic players. Media Psychol. 13, 339–363. doi: 10.1080/15213269.2010.524912

[ref13] Hartmann T. Vorderer P. (2010). It's okay to shoot a character: moral disengagement in violent video games. J. Commun. 60, 94–119. doi: 10.1111/j.1460-2466.2009.01459.x

[ref14] Heider F. (1958). *The psychology of interpersonal relations* . New Jersey: Lawrence Erlbaum Associates.

[ref15] İskender Ö. (2023). Identification with game characters: theoretical explanations, predictors, and psychological outcomes. Psikiyatride Güncel Yaklaşımlar 15, 203–219. doi: 10.18863/pgy.1104693

[ref16] Jicol C. Wan C. H. Doling B. Illingworth C. H. Yoon J. Headey C.. (2021). Effects of emotion and agency on presence in virtual reality. In Proceedings of the 2021 CHI conference on human factors in computing systems, Yokohama, Japan.

[ref17] Kanayama N. Hara M. Kimura K. (2021). Virtual reality alters cortical oscillations related to visuo-tactile integration during rubber hand illusion. Sci. Rep. 11:1436. doi: 10.1038/s41598-020-80807-y, PMID: 33446834 PMC7809445

[ref18] Kelley H. H. (1967). “Attribution theory in social psychology” in Nebraska symposium on motivation. ed. Levine D., vol. 15 (Lincoln: University of Nebraska Press), 192–238.

[ref19] Kilteni K. Groten R. Slater M. (2012). The sense of embodiment in virtual reality. Presence Teleop. Virt. 21, 373–387. doi: 10.1162/PRES_a_00124

[ref20] Klimmt C. Schmid H. Nosper A. Hartmann T. Vorderer P. (2006). How players manage moral concerns to make video game violence enjoyable. Communications 31, 309–328. doi: 10.1515/COMMUN.2006.020

[ref21] Lee K. M. (2004). Presence, explicated. Commun. Theory 14, 27–50. doi: 10.1111/j.1468-2885.2004.tb00302.x

[ref22] Lin S. F. (2011). Effect of opponent type on moral emotions and responses to video game play. Cyberpsychol. Behav. Soc. Netw. 14, 695–698. doi: 10.1089/cyber.2010.0523, PMID: 21557642

[ref23] Lombard M. Ditton T. (1997). At the heart of it all: the concept of presence. J. Comput.-Mediat. Commun. 3:JCMC321. doi: 10.1111/j.1083-6101.1997.tb00072.x

[ref24] Mahood C. Hanus M. (2017). Role-playing video games and emotion: how transportation into the narrative mediates the relationship between immoral actions and feelings of guilt. Psychol. Pop. Media Cult. 6, 61–73. doi: 10.1037/ppm0000084

[ref25] Makransky G. Lilleholt L. Aaby A. (2017). Development and validation of the multimodal presence scale for virtual reality environments: a confirmatory factor analysis and item response theory approach. Comput. Hum. Behav. 72, 276–285. doi: 10.1016/j.chb.2017.02.066

[ref26] Miller D. T. Ross M. (1975). Self-serving biases in the attribution of causality: fact or fiction? Psychol. Bull. 82, 213–225. doi: 10.1037/h0076486

[ref27] Morganti F. Marrakchi S. Urban P. P. Iannoccari G. A. Riva G. (2009). A virtual reality based tool for the assessment of “survey to route” spatial organization ability in elderly population: preliminary data. Cogn. Process. 10, 257–259. doi: 10.1007/s10339-009-0284-9, PMID: 19693582

[ref28] Nachez M. Schmoll P. (2022). Avatars, characters, multiple identities: Observations on dissociative processes. Hybrid: Revue des arts et médiations humaines, 9.

[ref29] O'Donnell T. (2005). Executioners, bystanders and victims: collective guilt, the legacy of denazification and the birth of twentieth-century transitional justice. Leg. Stud. 25, 627–667. doi: 10.1111/j.1748-121X.2005.tb00687.x

[ref30] Raney A. A. (2004). Expanding disposition theory: reconsidering character liking, moral evaluations, and enjoyment. Commun. Theory 14, 348–369. doi: 10.1111/j.1468-2885.2004.tb00319.x

[ref31] Raney A. A. (2006). “The psychology of disposition-based theories of media enjoyment” in Psychology of entertainment. eds. Bryant J. Vorderer P. (New Jersey: Lawrence Erlbaum Associates), 137–150.

[ref32] Raney A. A. (2011). Media enjoyment as a function of affective dispositions toward and moral judgment of characters. In Doveling K. Scheve C. von Konijn E. A. (Eds.), The Routledge handbook of emotions and mass media. (New York, NY, United States: Routledge), 166–178.

[ref33] Raney A. A. Janicke S. (2013). “How we enjoy and why we seek out morally complex characters in media entertainment” in Media and the moral mind. ed. Tamborini R. C. (New York, NY, United States: Routledge), 152–169.

[ref34] Sawilowsky S. (2009). New effect size rules of thumb. J. Mod. Appl. Stat. Methods 8, 597–599. doi: 10.22237/jmasm/1257035100

[ref35] Tauer J. M. Harackiewicz J. M. (1999). Winning isn't everything: competition, achievement orientation, and intrinsic motivation. J. Exp. Soc. Psychol. 35, 209–238. doi: 10.1006/jesp.1999.1383

[ref36] Weiner B. (2018). The legacy of an attribution approach to motivation and emotion: a no-crisis zone. Motiv. Sci. 4, 4–14. doi: 10.1037/mot0000082

[ref37] Zeller D. Friston K. J. Classen J. (2016). Dynamic causal modeling of touch-evoked potentials in the rubber hand illusion. NeuroImage 138, 266–273. doi: 10.1016/j.neuroimage.2016.05.065, PMID: 27241481

[ref38] Zillman D. Cantor J. R. (1977). Affective responses to the emotions of a protagonist. J. Exp. Soc. Psychol. 13, 155–165. doi: 10.1016/S0022-1031(77)80008-5

[ref39] Zillmann D. (2000). “Basal morality in drama appreciation” in Moving images, culture and the mind. ed. Bondebjerg I. (London, UK: University of Luton Press), 53–63.

[ref40] Zillmann D. (2013). “Dramaturgy for emotions from fictional narration” in Psychology of entertainment. ed. Bryant J. Vorderer P.. (New York, NY, United States: Routledge), 215–238.

[ref41] Zillmann D. Bryant J. (1975). Viewer’s moral sanction of retribution in the appreciation of dramatic presentations. J. Exp. Soc. Psychol. 11, 572–582. doi: 10.1016/0022-1031(75)90008-6

[ref42] Zillmann D. Cantor J. (1976). “A disposition theory of humor and mirth” in Humor and laughter: Theory, research, and application. eds. Chapman T. Foot H. (London: Wiley), 93–115.

